# The Measurement of Eye Movements in Mild Traumatic Brain Injury: A Structured Review of an Emerging Area

**DOI:** 10.3389/fspor.2020.00005

**Published:** 2020-01-28

**Authors:** Samuel Stuart, Lucy Parrington, Douglas Martini, Robert Peterka, James Chesnutt, Laurie King

**Affiliations:** ^1^Department of Sport, Exercise and Rehabilitation, Northumbria University, Newcastle upon Tyne, United Kingdom; ^2^Department of Neurology, Oregon Health and Science University, Portland, OR, United States; ^3^Veterans Affairs Portland Health Care System, Portland, OR, United States; ^4^National Center for Rehabilitative Auditory Research, Veterans Affairs Portland Health Care System, Portland, OR, United States; ^5^Department of Family Medicine, Oregon Health & Science University, Portland, OR, United States; ^6^Orthopaedics and Rehabilitation, Oregon Health & Science University, Portland, OR, United States

**Keywords:** mild traumatic brain injury, eye-tracking, eye movement, methods, vision

## Abstract

Mild traumatic brain injury (mTBI), or concussion, occurs following a direct or indirect force to the head that causes a change in brain function. Many neurological signs and symptoms of mTBI can be subtle and transient, and some can persist beyond the usual recovery timeframe, such as balance, cognitive or sensory disturbance that may pre-dispose to further injury in the future. There is currently no accepted definition or diagnostic criteria for mTBI and therefore no single assessment has been developed or accepted as being able to identify those with an mTBI. Eye-movement assessment may be useful, as specific eye-movements and their metrics can be attributed to specific brain regions or functions, and eye-movement involves a multitude of brain regions. Recently, research has focused on quantitative eye-movement assessments using eye-tracking technology for diagnosis and monitoring symptoms of an mTBI. However, the approaches taken to objectively measure eye-movements varies with respect to instrumentation, protocols and recognition of factors that may influence results, such as cognitive function or basic visual function. This review aimed to examine previous work that has measured eye-movements within those with mTBI to inform the development of robust or standardized testing protocols. Medline/PubMed, CINAHL, PsychInfo and Scopus databases were searched. Twenty-two articles met inclusion/exclusion criteria and were reviewed, which examined saccades, smooth pursuits, fixations and nystagmus in mTBI compared to controls. Current methodologies for data collection, analysis and interpretation from eye-tracking technology in individuals following an mTBI are discussed. In brief, a wide range of eye-movement instruments and outcome measures were reported, but validity and reliability of devices and metrics were insufficiently reported across studies. Interpretation of outcomes was complicated by poor study reporting of demographics, mTBI-related features (e.g., time since injury), and few studies considered the influence that cognitive or visual functions may have on eye-movements. The reviewed evidence suggests that eye-movements are impaired in mTBI, but future research is required to accurately and robustly establish findings. Standardization and reporting of eye-movement instruments, data collection procedures, processing algorithms and analysis methods are required. Recommendations also include comprehensive reporting of demographics, mTBI-related features, and confounding variables.

## Introduction

Eye movements are the basis of how humans gather information about the environment, which is then used to allow the perception of vital information needed for safe navigation or task performance. Eye movements have been investigated via various methods since the 1700s (Porterfield, 1752), with progression from eye-tracking that used large-scale photographic technology to invasive high resolution scleral search coils, and finally to more modern non-invasive small-scale infrared camera systems (Land, 2006). There are many eye movements that can be captured with modern technologies, such as saccades (fast eye movements), fixations (pauses on areas of interest), smooth pursuits (fixations on moving objects), and nystagmus (repetitive non-voluntary resetting eye movements; Tatler and Wade, 2003; Duchowski, 2007; Holmqvist et al., 2011). A combination of these eye movements provide the mechanisms through which we are able to explore and sample our environment (McPeek et al., 2000; Deubel and Schneider, 2003; Tatler and Wade, 2003; Marigold and Patla, 2008; Tatler, 2009; Stuart et al., 2014a). In order to derive and classify the different types of eye movements, a range of spatial-temporal and kinematic outcome variables are typically used, such as latency, velocity, acceleration, number/frequency, timing and duration (Duchowski, 2007; Holmqvist et al., 2011; Stuart et al., 2019a). Advancements in eye-tracking technologies have enabled eye movements to be monitored with small-scale devices that can be used in a variety of environments, such as research laboratories, clinics, field-based and community facilities. Similarly, collection of eye movement data with eye-tracking devices has progressed from traditional static tasks (e.g., seated or standing) to more dynamic tasks (e.g., walking or navigation of the environment), which is an important step toward understanding the impact that deficits can have on real-world function. The development of simple, high resolution, quantitative eye-tracking technologies is allowing disease or injury-specific impairments to be uncovered.

Eye movements are increasingly being studied in mild traumatic brain injury (mTBI) (commonly referred to as concussion; Thiagarajan et al., 2011; Ventura et al., 2015; Hunt et al., 2016; Snegireva et al., 2018), as eye-tracking protocols can be used to detect subtle deficits in cognitive, motor and visual processes that may occur following a head injury (Liversedge and Findlay, 2000; Maruta et al., 2010a). Detection of mTBI and monitoring of recovery of subtle impairments is not always possible with conventional means, such as neuroimaging (Eierud et al., 2014) or clinical assessments (McCrea et al., 2015). A lack of accurate and robust diagnostics, biomarkers and outcome measures leads to mTBI going undetected (Jeter et al., 2013; Kim et al., 2018; Quinones-Ossa et al., 2019). Undetected mTBI can lead to impaired functional activities, self-medication and return to sport/work/play before recovery is complete, which may lead to increased future injury risk and other health burden (McPherson et al., 2019; Reneker et al., 2019). Interestingly, the incidence of self-reported visual impairments in those following a traumatic brain injury has been reported to be as high as 90% (Ciuffreda et al., 2007), but incidence reports vary with the lowest recorded at 22% (Lara et al., 2001; Cockerham et al., 2009). This is not surprising, as the processing of vision and control of eye-movements is known to involve a large proportion of the brains circuits and regions (Antoniades et al., 2013), as well as underlying neural pathways and structures (Mays et al., 1986; Noda, 1991; Catz and Thier, 2007; Shinoda et al., 2019). For example, visual signals from the retina are sent to the superior colliculus then initially processed by the lateral geniculate nucleus, pulvina and mediordorsal thalamus, where signals are then sent for top-down visual processing at the pre-frontal cortex, frontal eye-field, supplementary eye field and lateral intraparietal area; as well as basic visual processing at the visual cortex (V1/V2, V4), middle temporal area and inferotemporal cortex, with the striatum, substantia nigra pars reticulate, and brainstem involved in eye movement initiation and control (Baluch and Itti, 2011). Therefore, as a result of an mTBI eye movements may be impaired and eye-movement recordings could provide a simple, quick and non-invasive means to quantify impairments and recovery in mTBI (Snegireva et al., 2019). Furthermore, imaging evidence suggests that saccades, smooth pursuits and nystagmus eye movements activate largely similar neural structures (Konen et al., 2005; Dieterich et al., 2009), and therefore individual eye movement tests may be of value to mTBI diagnosis.

Eye-tracking technology has been used to further understand mTBI-related impairments in eye-movements (Akhand et al., 2019), demonstrating some efficacy for use in mTBI assessment and clinical diagnostics. However, until recently most eye movement research in mTBI involved simple, subjective, clinical, self-reported, or symptom-based tasks that could be performed in the field or within clinic with minimal training [e.g., the vestibular/ocular-motor screening (VOMS) Mucha et al., 2014]. Several previous reviews of studies that have used self-report/symptom-based outcomes have reported vision impairments in mTBI (Thiagarajan et al., 2011; Hunt et al., 2016; Whitney and Sparto, 2019). However, many mTBI subjective rating scales have not had rigorous validity or reliability testing (Alla et al., 2009), and scales may miss subtle symptoms due to reliance on clinician experience and self-report (Meier et al., 2015). Progression to the use of eye-tracking devices in research that are capable of capturing eye-movements at high speed and providing quantifiable outcomes has led to an array of testing protocols (Hunt et al., 2016; Snegireva et al., 2018), indicating a lack of standardization that limits outcome interpretation and generalizability. Several recent reviews provided overviews of reported impairment of eye-movement outcome measures in mTBI (Hunt et al., 2016; Snegireva et al., 2018), but provided limited details regarding the use of specific eye-tracking methodologies and how differences in methods or devices may impact findings. Researchers who want to conduct similar research are therefore left with the choice between numerous eye-tracking devices, outcomes and protocols that differ in many respects and complexities. In the process of developing robust protocols it is helpful to have evidence-based recommendations. We therefore examined previous work that assessed eye movements in mTBI and healthy control participants in order to provide some guidance regarding the selection of appropriate methodology.

We focused the review on the following: (1) eye-tracking instrumentation used to examine people following an mTBI compared to healthy controls; (2) commonly reported eye movement outcomes from eye-tracking studies; (3) mTBI specific influences on these eye movement outcomes; and (4) recommendations concerning future protocols.

## Methods

### Search Strategy

**Figure 1:**
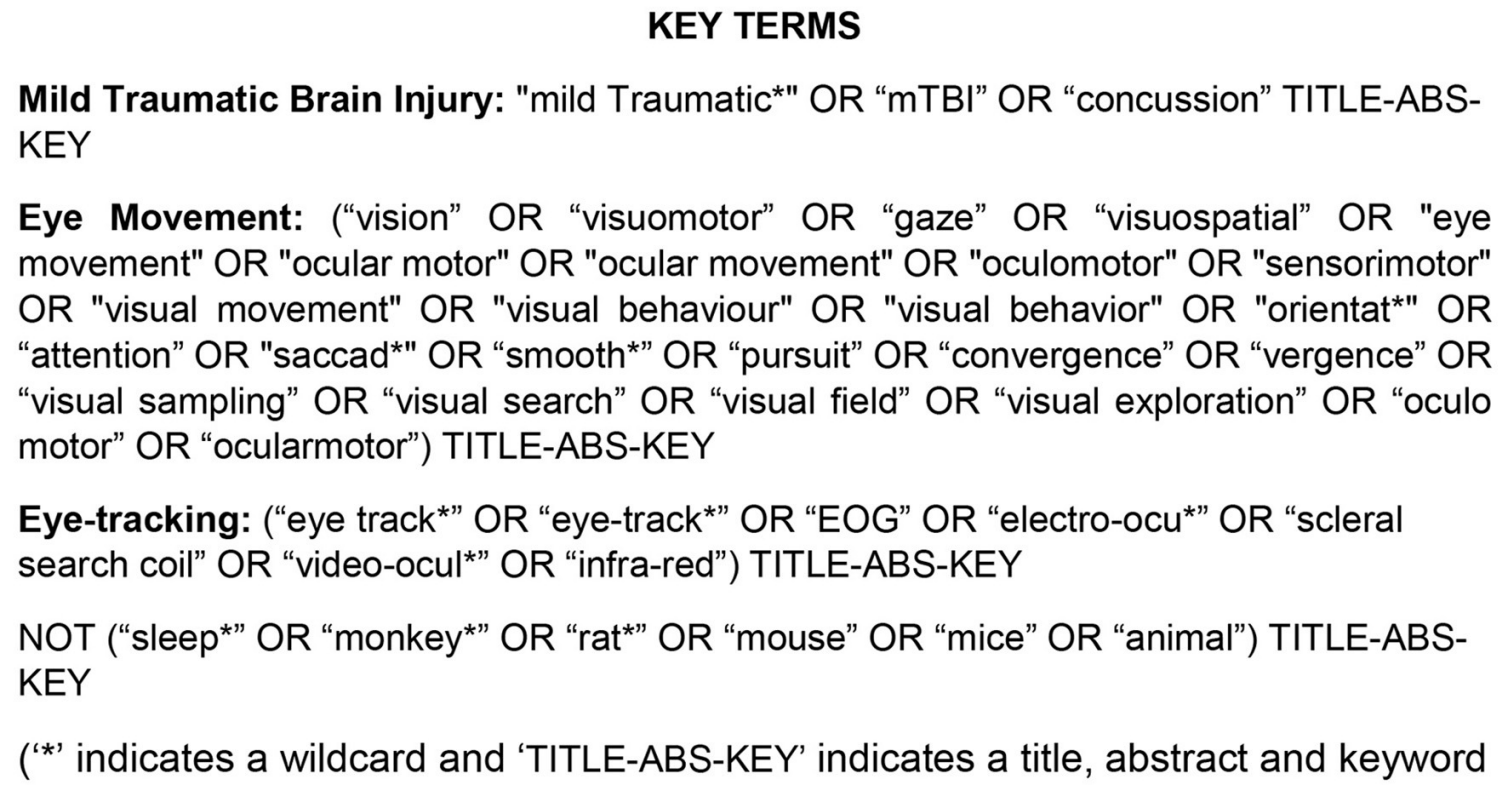
Search strategy used to screen for relevant articles included in this review.

The key terms were “mild Traumatic Brain Injury,” “Eye movement,” and “Eye-tracking.” A list of synonyms was created for each key term (Figure 1). Key terms were matched and expanded with medical subject headings (MeSH) in each separate database where appropriate. Databases searched included Medline/Pubmed (from 1950), PsychInfo (from 1806), CINAHL (from 1937), and Scopus to July 2019. Studies were relevant if they used terminology that focused on eye movement tracking in those with mTBI and healthy control subjects in the title, abstract or keywords. Articles with titles related to “sleep,” “monkeys,” “rats,” “mice,” or “animal” models were excluded using separate key terms.

An initial title screen for relevant articles was performed by the reviewer (SS) once the searched database results had been combined. After the initial title screen, both the titles and abstracts of the selected articles were reviewed by three independent reviewers (SS, DM, LP). A review of the full text was required if it was not clear from the title or abstract whether the study met the review criteria.

### Inclusion and Exclusion Criteria

Articles were included if they reported use of an eye-tracking measurement instrument to quantify eye movements (i.e., saccades, smooth pursuits, convergence, fixations etc.) in people with mTBI. Studies were included only if they tested a healthy control cohort or used a baseline pre-injury test as a control for comparison with mTBI cohorts so that injury-specific differences could be identified. If articles including another clinical cohort (i.e., whiplash, acquired brain injury, moderate to severe TBI; Samadani et al., 2015), or an additional visual assessment that was not quantified via eye-tracking (e.g., measurement of convergence), only the eye-tracking data from the mTBI and healthy control cohorts was reviewed. If clinical cohorts were combined [i.e., mTBI with traumatic whiplash (Herishanu, 1992) or mild-to-severe TBI (Vakil et al., 2019)] or studies used a control group that had experienced an mTBI and were classified as recovered compared to a symptomatic mTBI population (Heitger et al., 2009), then the data were not reviewed. Acute and chronic (including post-mTBI syndrome) mTBI cohorts were reviewed, but studies without a diagnosis of an mTBI [i.e., repetitive head injuries or those with a self-reported history of head injuries with no current symptoms or recent referral to study by a clinician (Rizzo et al., 2016)] were excluded. Rehabilitation studies that did not include a baseline examination, or did not include any cross-sectional comparison to healthy controls were not reviewed (Kaldoja et al., 2015; Johansson et al., 2017). Only articles written in English were considered for review and any abstracts, case studies, reviews, book chapters, commentaries, discussion papers, editorials or conference proceedings were excluded.

### Data Extraction

Data were extracted by the reviewer (SS) and were synthesized into table format by the reviewer (SS) and a second reviewer (LP) confirmed the entered data (Tables 1–3). Data included demographic, eye movement measurement instruments, eye movement outcomes, study protocol and key findings.

**Table 1:** Participant characteristics, mTBI diagnosis or definition, inclusion, and exclusion criteria.

**References**	**Participants**	**mTBI diagnosis or definition**	**Inclusion criteria**	**Exclusion criteria**
Balaban et al. (2016)	100 acute mTBI – Aged 26.4 ± 6.7 years – 33 female/67 male – 62.4 ± 37.6 h since injury200 Healthy Controls – Aged 28.0 ± 6.1 years – 44 female/156 male – Recruitment of mTBI from emergency rooms at civilian and military hospitals. Controls recruited from study location sites	– Diagnosis of mTBI from an emergency room staff physician – Head injury with a Glasgow Comma Scale of 14 or greater with no loss of consciousness >30 min	– Aged 18-45 years old – Within 6 days of injury Control specific; – No active medical condition – No history of significant mTBI – No ear or balance disorders	NR
Cifu et al. (2015)	60 Chronic post-concussion symptoms mTBI – Aged 23.2 ± 3.0 years – 8.5 ± 6.6 months since injury 26 Healthy controls – Recruitment from military. Controls recruited from an academic military center.	– TBI confirmed by a physiatrist following referral – Ongoing post-concussion symptoms evidenced with Rivermead Post Concussion Symptom Questionnaire	NR	– History of prior neurologic – Ophthalmologic – Other health conditions, including whether they had any subjective visual complaints, such as blurred vision, double vision, or floaters
Cochrane et al. (2019)	28 mTBI – Aged 20.7 ± 1.9 years – 11 female/17 male 87 Healthy controls – Aged 20.6 ± 1.8 years – 39 female/48 male – 23 reported mTBI history but with no current symptoms – Recruitment from community, university students who performed recreational sports and US Division 1 university men's football and women's soccer teams	NR	– Tested within 72 h to 2 weeks post-injury – Aged 18–24 years old – Maximum 2 weeks since mTBI – Active in sport	NR
Contreras et al. (2011)	12 Chronic post-mTBI symptoms – Aged 29.7 ± 7.3 years – 2.2 ± 1.8 years since injury 12 Healthy controls – Aged 27.9 ± 4.9 years – Recruitment NR	NR	– Head injuries limited to one – Blunt, isolated mTBI – No presence of posttraumatic amnesia – No cranial nerve abnormalities (except those affecting the sense of smell) – Non-intoxication	– Previous mTBI with loss of consciousness for periods longer than 24 h, – History of multiple mTBI with loss of consciousness – Pregnancy – History of drug or alcohol abuse – Pre-injury neurological or psychiatric diagnosis of an axis I or axis II disorder – General anesthesia within two weeks before testing – Seizure following trauma – Seizure disorders – Pre-injury use of psychotropic medication(s)Control specific: – No history of head injury or head trauma – Non-intoxication
DiCesare et al. (2017)	17 mTBI – Aged 16.8 ± 1.2 years – 5 females/12 males – 7.7 ± 4.7 Days since injury 17 Healthy controls – Aged 16.8 ± 0.7 – 7 females/10 males –Recruitment NR	– Recently experienced and diagnosed with mTBI (missing exact dates from 2 subjects)	NR	NR
Diwakar et al. (2015)	25 Chronic post-concussion symptoms mTBI – Aged 32.7 ± 11.2 years – 84% males – 31.8 ± 18.3 months since injury 25 Healthy Controls – Aged 31.8 ± 10.6 – 68% males–Recruitment from university TBI clinics and studies, as well as community. Controls recruited from community and other studies.	– Persistent symptoms since mTBI	– A single TBI with or without loss of consciousness within 3 months to 5.5 years prior to testing – Any persistent PCS symptoms – A normal CT or MRI for patients who went to the emergency room – A Glasgow Coma Scale (GCS) of 13–15 at time of injury, if available.	– Hospitalized for their injury – Were intubated – Had multiple TBIs – Had loss of job due to the injury – Confirmed use of psychotropic or cognitive enhancing medication – Showed evidence of malingering on the Test of Memory Malingering (i.e., cut-off score below 45 on trial 2)All subjects: – Neurological diagnosis other than mTBI – History of post-traumatic stress disorder – Neurological disorders other than TBI (e.g., seizure disorder) – Pre-morbid major psychiatric disorders (e.g., major depressive disorder) – Alcoholism or substance abuse – Attention deficit hyperactivity disorder (ADHD).
Hecimovich et al. (2019)	19 Subjects at baseline – Aged 13.9 ± 0.3 years – 0 female/19 male 6 post-injury mTBI – Recruited from Australian Rules Football Players	– Impact to the head either witnessed during the game or retrospective identified using a log book recording – Head impact was documented in log book if a subject had player-to-player or player-to-surface head contact, or whiplash-like head movement, or self-reported symptoms	NR	– A history of concussion or traumatic brain injury – A history of neurological or ophthalmological disease (other than refractive error) – A history of concussion – Neurological impairment – Learning disability – Visual dysfunction – Taking central nervous system-active medications
Hoffer et al. (2017)	106 acute mTBI – Aged 26.2 ± 6.5 years – 34 female/72 male – 64.8 ± 39.3 h since injury Follow-up at 7-10 days and 14-17 days to examine sub-acute injury period 300 Healthy controls – Aged 27.5 ± 6.5 years – 95 female/205 male – Recruitment of mTBI from emergency rooms at civilian and military hospitals. Controls recruited from study location sites	– Diagnosis of mTBI from an emergency room staff physician – Head injury with neurosensory sequelae and a Glasgow Comma Scale of 14 or greater with no loss of consciousness >30 min – Neurosensory symptoms included but were not limited to dizziness, hearing loss, headache, cognitive difficulties, and sleep disorders	– Aged 18-45 years old – Within 6 days of injury – No head injury 12 months prior to current injury – Never hospitalized for a head injury Control specific; – No active medical condition – No history of significant mTBI – No ear or balance disorders	– NR
Howell et al. (2018)	44 mTBI – Aged 14.1 ± 2.2 years – 17 female/27 male – 6.4 ± 2.5 Days since injury 35 Health Controls – Aged 14.3 ± 2.4 years – 20 female/15 male –Recruitment from two sport concussion clinics of regional children's hospital, and controls from hospital employees	– Sports medicine physician diagnosed mTBI – Defined as “a direct blow to the head, face, neck, or elsewhere on the body, resulting in the rapid onset of impairment of neurologic function”	– Within 10 days of mTBI – Between ages 8 and 18 years – mTBI via sports or mechanism involving forces similar to sports (e.g., falling from level ground, recreational activity injury)	– Concurrent injury sustained at the time of concussion – History of permanent memory loss – Significant sensory deficits (e.g., deafness or blindness) – A history of psychiatric disorders – Falling from height – Motor vehicle collisionControl specific: – Diagnosed concussion within the year prior to testing – A concurrent injury limiting sport participation – History of permanent memory loss, significant sensory deficits (e.g., deafness or blindness) – History of psychiatric disorders
Johnson et al. (2015a)	9 acute mTBI 7 follow-up sub-acute mTBI – Aged 18-21 years – 3 female/4 male 9 Healthy Controls–Recruitment NR	NR	– Within 7 days of injury for acute mTBI – At 30 days for sub-acute mTBI	NR
Johnson et al. (2015b)	9 mTBI – Age range 18-21 years – 3 female/6 male 9 Healthy Controls – Age range 20-22 years – 3 female/6 male – Recruitment from Sport Concussion Program at University	NR	– Within 7 days of injury	– History of psychiatric or neurological disorders,–On any current medications
Kelly et al. (2019)	50 mTBI – Aged 15.2 (range 13-18) years – 24 female/26 male – Days since injury 22.1 (range 1 to 328) 170 Healthy Controls – Aged 15.5 (range 11–18) years – Recruitment from high-school aged athletes	– mTBI diagnosis confirmed by director of Sports Medicine Concussion Clinic (or by a neurologist in Neurology Concussion Clinic) – Defined as “a transient alteration of normal brain function typically affecting orientation and memory due to an external mechanical force, which may have involved loss of consciousness; concussion was considered equivalent to mTBI”	– Ongoing mTBI symptoms – Males and females – Aged 13 to 18 years old – Able and willing to assent or consent; a parent or legal guardian provided consent for those under 18 years old	– Brain injury resulting from a penetrating wound to the head, neck, face, or brai – History of schizophrenia or major depression – Previous concussion with incomplete symptom recovery
Maruta et al. (2010b)	17 Chronic post-mTBI symptoms – 7 females/10 males – 2.7 years since injury (range 6 weeks to 5 years) 9 Healthy Controls – Age range 19 to 31 years – 3 females/6 males – Recruitment from local concussion clinics and community.	NR	– Blunt, isolated TBI, posttraumatic amnesia, and a Glasgow Coma Scale score of 13 to 15 at time of injury.	– Pregnancy – A history of neurological or psychiatric diagnosis – A history of seizure (before the injury) – A history of drug or alcohol abuse
Maruta et al. (2016)	33 Chronic post-mTBI symptoms – Aged 34.9 ± 14.0 years 16 male, 17 female – 1.6 years since injury (range 4 months to 4.5 years) 140 Healthy Controls – Aged 36.6 ± 10.6 years 67 male, 73 female – Recruitment from health professional, university and community. Also, Brain Trauma Foundation website and newsletters from local brain injury organizations.	NR	– Males and females – At least 12 years of education – Aged 18 to 55 years old mTBI specific: – persistent problems believed to result from an isolated concussive head injury that occurred between 90 days and 5 years prior to the date of neurocognitive testing – Documented medical attention at the time of injury; PTA at the time of injury – A complete BISQ – If an LOC occurred, it did not exceed 24 h in the period following the injury. Control specific: – Have had a T-score <75 on the CAARS-S:S – A score <16 on the CES-D – A negative BISQ outcome	– A history of gross vision or hearing problems – A history of a substance abuse – A history of a neurological or psychiatric disorder – General anesthesia within the 14 days prior to neurocognitive testing – Current use of a psychotropic medication – Current pregnancy.Control specific: – Any history of a confirmed concussive head injury or BISQ-identified injury was exclusionarymTBI specific: – A history of prior concussive head injury was exclusionary only if it resulted in an emergency department visit that required conventional neuroimaging – Seizures – Other medical problems
Maruta et al. (2017)	43 Chronic post-mTBI symptoms – 22 female/21 male 5 Acute mTBI – 3 female/2 male 140 Healthy Controls – 74 female/66 male – Recruitment NR	NR	Chronic mTBI specific; – Persistent symptoms following an mTBI that occurred 90 days to 5 years before date of testing – Had post-traumatic amnesia at the time of injury – Had a loss of consciousness not exceeding 24 h in the period following the injury Acute mTBI specific; – Within 2 weeks post-injury All Subjects; – Aged 18 to 55 years old – At least 12 years of education	– Pregnant – History of drug or alcohol abuse – Neurological or psychiatric illness, or seizure.
Maruta et al. (2018)	29 mTBI – Aged 18.4 ± 2.3 years – 14 female/15 male – 5.3 ± 3.3 Days since injury – 2.8 ± 2.5 months after baseline testing 1,442 baseline tested – Recruitment from local school, university and community athletic organizations	– A diagnosis by a physician was not required. Prospective acute post-concussion enrolment was based on inclusion criteria consisting of an experience within 2 weeks of a concussion that resulted in loss of consciousness, post-traumatic amnesia, dizziness, nausea, headaches, balance problems, blurred or double vision, or daze and confusion, and on an exclusion criterion of intoxication at the time of injury.	– Participation in organized competitive athletic activity – Aged 12–30 years – Normal or corrected to normal vision, For athletes over the age 18: – A high school diploma or equivalent, or expected timely high school graduation	– A prior history of traumatic brain injury (including concussion) – Alcohol or substance abuse – A known neurologic disorder, – A psychiatric condition previously known or identified using questionnaires for attention deficit hyperactivity disorder – Depression – Anxiety disorders – A known vision-related disease or abnormality
Murray et al. (2014)	9 mTBI – Aged 16.0 ± 3.0 years – 7 female/2 male 9 Healthy controls – Aged 24.3 ±7.5 years – 6 female/3 male – Recruitment from concussion management clinics	– Diagnosed by athletic trainer or physician	– Tested 48-72 h post injury	– Abnormal behavior (expressed by an extreme emotional state) – Excessive neurological symptoms (indication of a traumatic brain injury) – The inability to safely conduct the experiment due to major bodily injury such as lacerations, bone fractures or the like
Murray et al. (2017)	10 mTBI – Aged 18.9 years – 4 female/6 male 10 Healthy Controls – Aged 18.3 years – 4 female/6 male – Recruitment from unspecified athletic population	– Diagnosed by an athletic trainer or physician	– Tested within 48 h post injury Control Specific: – Tested prior to the beginning of their respective athletic season.	– Free of any musculoskeletal and/or neuromuscular injury beyond the documented concussion injury – Had no history of psychiatric illness, Attention Deficit Hyperactivity Disorder and/or seizures – Had no documented concussion within the past 6 months as determined by self-report
Stuart et al. (2019b)	10 mTBI – Aged 30.1 ± 12.8 years – 8 female/2 male – 39.5 ± 21.7 Days since injury 10 Healthy Controls – Aged 26.3 ± 5.2 years – 8 female/2 male – Recruitment NR	– Diagnosed by a physician – Defined with following criteria “no CT scan (or a normal CT scan if obtained), no loss of consciousness exceeding 30 min, no alteration of consciousness/mental state up to 24 h post-injury, and no post-traumatic amnesia that exceeded one day”	– A diagnosis of mTBI within 12 weeks; the mechanism of injury was not be restricted, so may include whiplash if subjects passed a cervical screen. – Aged between 18–60 years old. – SCAT5 symptom evaluation sub–score ≥1 for balance, dizziness nausea, headache or vision AND a minimum total score of 15. – No or minimal cognitive impairment having ≤ 9 on the Short Blessed Test	– Other musculoskeletal, neurological, or sensory deficits that could explain dysfunction – Moderate to severe substance-use disorder within the past month (American Psychiatric Association 2013) – Severe pain during an initial clinical evaluation ≥7/10 subjective rating) – Current pregnancy – Unable to abstain from medications that might impair balance 24 h before testing – Contraindications to rehabilitation such as unstable c-spine – Active participation in physical therapy for their concussion, however participants could be undertaking other forms of treatment for their symptoms such as massage, acupuncture, and counseling
Suh et al. (2006)	20 Chronic mTBI – Aged 38.0 ± 11.2 years – 6 weeks–24 months post injury 6 Acute mTBI – Aged 34.2 ± 14.3 years – 8–12 Days post injury 26 Healthy Controls – Aged 30.9 ± 13.0 years – Recruitment NR	– Glasgow Coma Scale (GCS) score 13–15 at time of injury – Aged 18–60 years	– Blunt, isolated TBI – Post-traumatic amnesia (PTA) – Non-intoxication – Normal of corrected to normal vision Chronic mTBI specific; – Within 2 years post injury Acute mTBI specific; – Within 14 days post injury Control specific; – No prior history of TBI	– Multiple TBI with loss of consciousness (LOC), – Pregnancy – Drug or alcohol abuse – Neurological or psychiatric diagnosis, or seizures – General anesthesia within two weeks following trauma
Webb et al. (2018)	15 mTBI – Aged range 21–26 years – 4 female/11 male – Within 2–6 days since injury 15 Healthy Controls – Age and sex matched to mTBI group	– Clinical judgement of physician and physician assistant – SCAT 3 score	– Right handed – Normal or corrected to normal vision	Control specific – Previous or current diagnosis of a neurological or neuropsychiatric deficit (including a mTBI), attention deficit hyperactivity disorder, or a documented learning impairment – Same criteria for mTBI group, except current mTBI diagnosis – History of self-reported previous mTBI
Wetzel et al. (2018)	71 Chronic post-mTBI symptoms – Aged 33.0 ± 7.0 – 1 female/70 male – 28% had injury 3 months to 1 year before study 75 Healthy controls – Aged 39.0 ± 13.0 – 17 female/58 male – Recruitment from military and community	NR	– Aged 18 to 65 years old – Persistent symptoms following an mTBI 3 months to 5 years prior to testing – Head injury caused by non-penetrating trauma or blast exposure; and resulted in a period of loss of, or a decreased level of, consciousness (up to 30 min), a loss of memory for events immediately before or after the injury (up to 24 h), or alteration in mental state at the time of the injury (becoming dazed or confused)	mTBI specific; – Individuals with contraindications to hyperbaric pressurization and HBO2, conditions that might confound outcome measures such as refractive eye surgery within 90 days prior to enrolment, or life experiences that might expose study blindingControl specific; – Known history of brain injury – Diagnosis of neurologic disorders – Active therapy for affective disorder – Behavioral disorder – Psychologic disorders – Diabetes – Chronic migraines – Headaches – Dizziness – History of combat – Post-traumatic stress disorder (PTSD) – Prescription drug use known to impact neurologic function – Atrial septal defects – Developmental delays – Habitual use of cannabis or history of illicit drug or alcohol abuse – Binocular vision not correctable to 20/50 – Deafness – Active malignancy.

*NR, Not Reported; EOG, Electro-oculography; mTBI: mild Traumatic Brain Injury; TBI, traumatic brain injury; HC, Healthy control; Data are presented as means ± standard deviation unless otherwise stated*.

## Results

### The Evidence Base

**Figure 2:**
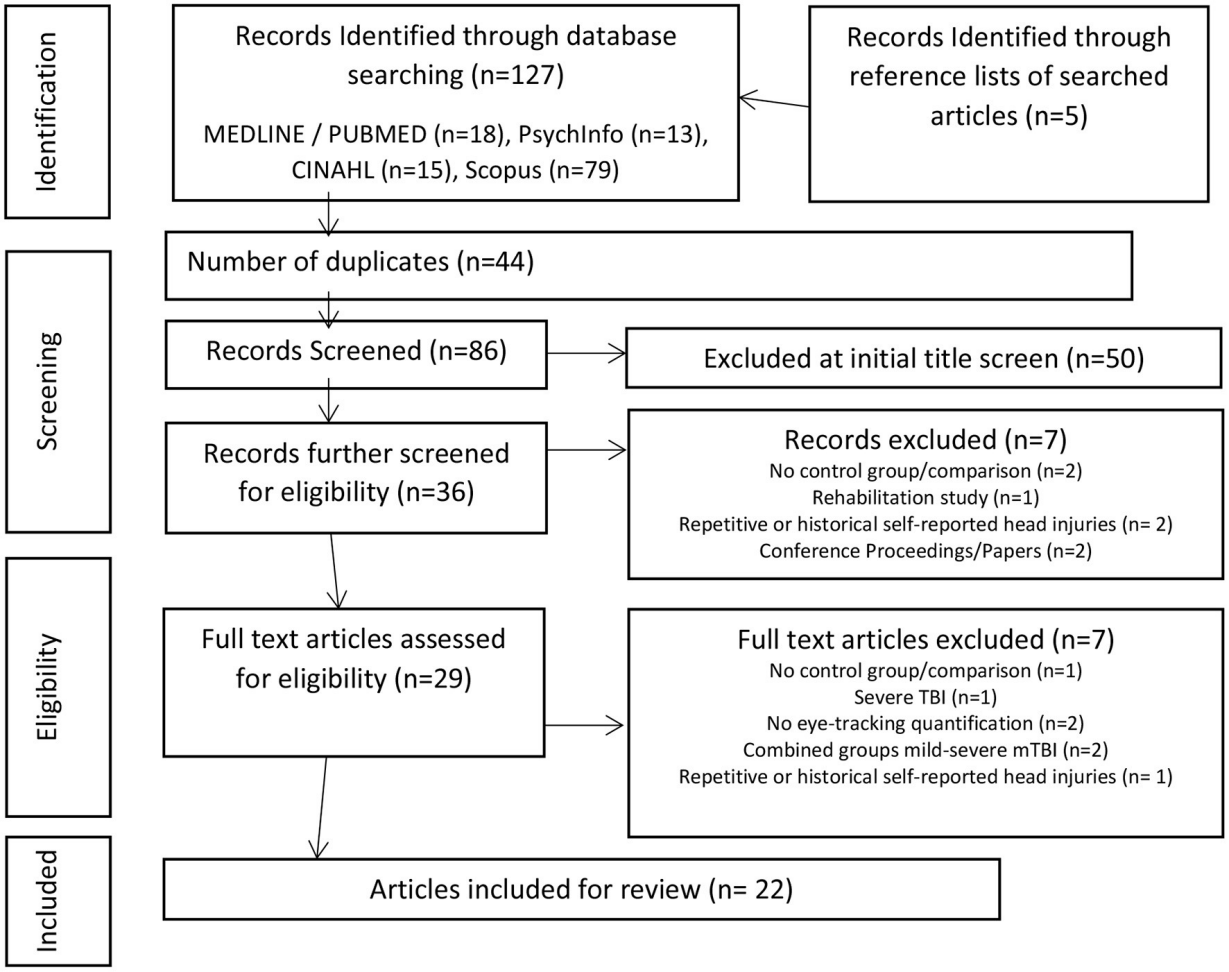
PRISMA flow chart of study design (Adapted from Moher et al., 2009).

The search strategy yielded 86 articles, excluding duplicates (Figure 2–adapted from Moher et al., 2009). There was an initial screening in 128 articles of interest of which 22 were identified for inclusion for review by consensus of the screening reviewers (SS, LP, DM). Of the title screened 50 were excluded for not meeting inclusion criteria of the review. The majority of screened studies were excluded because they were either not relevant or did not provide quantitative measurement of eye movements in mTBI (Figure 2).

### Participants

The reviewed articles (*n* = 22) investigated healthy controls and mTBI with average age ranges between 13 and 39 years old (Table 1), with the majority of the studies including both males and females. Several studies did not provide specific demographic characteristics of participants, such as age (Maruta et al., 2010b; Johnson et al., 2015a; Webb et al., 2018), sex (Suh et al., 2006; Contreras et al., 2011; Cifu et al., 2015), time since injury (Johnson et al., 2015a,b; Maruta et al., 2017; Webb et al., 2018; Cochrane et al., 2019) etc. There were also various inclusion and exclusion criteria for participants within the reviewed studies, with little consensus and a lack of reporting in some studies. Seven of the studies examined subjects with chronic post-mTBI symptoms (time since injury ranged from 3 months to 5 years) and 13 studies examined subjects with acute/sub-acute mTBI (time since injury ranged from within 2–40 days post-injury, Table 1). The majority of the studies compared mTBI to healthy controls, but two studies (Maruta et al., 2018; Hecimovich et al., 2019) examined athletes during a pre-season baseline and then a follow-up post-injury. One study (Kelly et al., 2019) investigated mTBI subjects at an average of 22.2 days post-injury, but time since injury ranged from 1 to 328 days, so both acute and chronic mTBI subjects were included as one cohort. Similarly, two other studies grouped acute and chronic mTBI for all of their data analysis (Suh et al., 2006; Wetzel et al., 2018). Several studies also examined the same mTBI and control cohorts but produced one article on the baseline acute injury testing and another on the follow-up sub-acute periods (Johnson et al., 2015a,b; Balaban et al., 2016; Hoffer et al., 2017).

### Instruments

Eye movements in the reviewed articles were measured using a variety of eye-tracking instruments, which largely depended on the desired eye-movement outcome or task being evaluated. For example, stationary eye-trackers were used for activities where restricted head movement was required, whereas mobile eye-trackers tended to be used for tasks that allowed head movement (i.e., walking or playing a computer game while standing balance was also examined; Murray et al., 2014, 2017; Stuart et al., 2019b). The 22 articles described an array of instrumentation including desk or computer-mounted infrared eye-trackers, rotary chairs within enclosed rooms, tethered head-mounted eye-trackers and fully mobile eye-trackers (Table 2). The sampling frequencies used to record eye movements varied considerably, despite many studies using similar devices (frequency range 60–1,000 Hz, Table 2). One study (Hecimovich et al., 2019) using the King-Devick eye-tracking system did not report the sampling frequency of the device.

**Table 2:** Study Protocol, eye movement instrument, outcome measures, and definitions.

**References**	**Test protocol**	**Eye movement instrument**	**Eye movement outcome measures**	**Eye movement outcome definition**
Balaban et al. (2016)	Static/seated	I-Portal Neuro Otologic Test Center (Neuro kinetics Inc.) – 100 Hz – Infra-red – Binocular	– Saccades – Random saccades – Anti-saccades – Predictive saccades – Self-paced saccades – Smooth Pursuit – Optokinetic Nystagmus – Gaze horizontal	NR
Cifu et al. (2015)	Static/seated	Eyelink II (SR Research) – 500 Hz – Binocular – 3 point calibration	– Saccades – Fixation – Smooth Pursuit	Saccade – Amplitude >0.1° – Velocity >20°/s – Acceleration >400°/s^2^ Fixation – When eye relatively stable – Low velocity – Low acceleration – No Directional trend Smooth Pursuit – Movement failed to meet saccadic inclusion criteria – Velocity greater than a fixation – Acceleration less than a saccade – Velocity and direction of eye movement closely matches target
Cochrane et al. (2019)	Static/seated	I-Portal Neuro Otologic Test Center chair system (Neuro Kinetics Inc.) – Binocular – 100 Hz – Infra-red	– Saccades – Smooth Pursuit – Optokinetic Nystagmus	NR
Contreras et al. (2011)	Static/seated	Eyelink II (SR Research) – 500 Hz – Binocular – Infra-red – 9 point calibration	Smooth Pursuit	Saccade – Velocity >29°/s – Acceleration >573°/s^2^ – Duration 20–240 ms Smooth Pursuit – Saccades removed
DiCesare et al. (2017)	Static/seated	Tobii X2-60 Eye Tracker (Tobii) – 60 Hz – 5 point calibration	– Saccades – Fixation – Smooth Pursuit	Saccade – Velocity >30°/sFixation – Velocity <30°/s
Diwakar et al. (2015)	Static/seated	Eyelink 1000 (SR Research) – NR	Smooth Pursuit	Saccade – Velocity >100°/s – Acceleration >1,500°/s^2^
Hecimovich et al. (2019)	Static/seated	K-D Eye Tracking System, EyeTech VT3 Mini (EyeTech Digital Systems) – Infrared	– Saccades – Blinks	NR
Hoffer et al. (2017)	Static/seated	I-Portal Neuro Otologic Test Center (Neuro kinetics Inc.) – 100 Hz – Infra-red – Binocular	– Saccades – Random Saccades – Anti-saccades – Predictive saccades – Self-paced saccades – Smooth Pursuit – Optokinetic Nystagmus – Gaze horizontal	NR
Howell et al. (2018)	Static/seated	Eyelink 1000 (SR Research) – 500 Hz – Analyzed with commercial software (Oculogica, Inc.)	– Eye skew – Normalized eye skew – Eye movement variance ratio – Eye distance	NR
Johnson et al. (2015a)	Lying down in MRI machine	ViewPoint Eye-Tracker (Arrington Research, Inc.) – 60 Hz – 16 point calibration – Analyzed with custom MATLAB codes	– Saccades – Anti-saccade – Self-paced saccade – Memory-guided saccade	NR
Johnson et al. (2015b)	Lying down in MRI machine	Viewpoint Eye-tracker MRI compatible eye tracking system (PC-60, Arrington Research, Inc.) – 60 Hz – Integrated into VisuaStim Digital Goggles	– Saccades – Reflexive saccades – Anti-saccades – Memory guided saccades – Self-paced saccades – Smooth Pursuit – Fixation	NR
Kelly et al. (2019)	Static/seated	Video Nystagmograph (VNG) (I-Portal) – 100 Hz	– Smooth Pursuit – Saccades – Predictive saccades – Anti-saccades – Optokinetic Nystagmus	NR
Maruta et al. (2010b)	Static/seated	Eye link II (SR Research) – 500 Hz – 9 point calibration – Analysis with custom MATLAB algorithms	Smooth Pursuit	Saccade – Velocity >100 °/s – Acceleration >1,500 °/s^2^Smooth Pursuit – Saccadic intrusions identified and removed (e.g., blinks)
Maruta et al. (2016)	Static/seated	Eyelink 1000 (SR Research) – NR; referred to previous methods	Smooth Pursuit	NR
Maruta et al. (2017)	Static/seated	Eyelink CL (SR Research) – NR; referred to previous methods	Smooth Pursuit	Smooth Pursuit – Saccadic intrusions identified and removed (e.g., blinks)
Maruta et al. (2018)	Static/seated	Eyelink 1000 (SR Research) – NR	Smooth Pursuit	Smooth Pursuit – Saccadic intrusions identified and removed (e.g., blinks)
Murray et al. (2014)	Dynamic/Standing	ASL Eye Tracking system (model H6, Applied Science Laboratories) – 120 Hz – Tethered system – Monocular (left eye only) – 9 point calibration	Gaze stabilization	NR
Murray et al. (2017)	Dynamic/Standing	ASL Eye Tracking system (model H7, Applied Science Laboratories) – 240 Hz – Tethered system – Monocular (left eye only)	Saccades	Fixation – 25 consecutive frames (gaze points)
Stuart et al. (2019b)	Dynamic/Walking	Tobii Pro Glasses 2 (Tobii Technology, Inc.) – 100 Hz – Head-mounted and mobile – Binocular – Analyzed using custom MATLAB algorithm – 1 point calibration	Saccades	Saccade – Velocity >240°/s – Acceleration >3,000 °/s^2^ – Duration <100 ms – Distance >5° – Blinks identified and removed Fixation – Velocity <240 °/s – Acceleration <3,000 °/s^2^Duration – >100 ms
Suh et al. (2006)	Static/seated	Eyelink II – 500 Hz – Infrared – 9 point calibration	Smooth Pursuit	Saccade – Velocity >40°/s Smooth Pursuit – Saccades identified and removed
Webb et al. (2018)	Static/seated	Eye-Trac6 (Applied Sciences Laboratories) – 360 Hz – 9 point calibration performed twice – Left eye only – Video-based eye-tracker	– Anti-saccades – Pro-saccades	Saccade – Velocity >30°/s – Acceleration >8,000°/s^2^ – Duration <42 ms
Wetzel et al. (2018)	Static/seated	Eyelink 1000 (SR Research) – 9 point calibration – 500 Hz	– Saccades – Self-paced saccades (reading) – Memory guided saccades – Anti-saccades – Smooth Pursuit – Fixation	Saccade – Velocity >20°/s – Acceleration >400°/s^2^ – Distance >0.1° Smooth Pursuit – Velocity >30°/s – Acceleration >2,000°/s^2^

*NR denotes not reported, mTBI, mild traumatic brain injury, s, seconds*.

### Reliability and Validity

Of particular importance was that none of the reviewed studies reported the validity or reliability of the eye-tracking instrumentation used, and studies provided no detail regarding manufacturer specifications of the equipment (i.e., accuracy of tracking).

There was also very poor reporting of specific data processing or analysis methods used to derive the outcomes of interest within the reviewed studies. Only one study comprehensively provided their eye-tracking data processing method to derive eye movement outcomes, as the article developed and validated an eye-tracker algorithm to derive saccades while walking in mTBI and controls (Stuart et al., 2019b). Whereas another study reported that if there was <70% of eye-tracking data available then their instrumentation stated the trial was not valid and it was repeated until a valid trial was collected (Cochrane et al., 2019). One study also reported a comparison of their smooth pursuit synchronization index outcome with a traditional measure of velocity error to validate their outcome measure (Contreras et al., 2011). One study used commercial software (Oculogica, Inc.) to process their data (Howell et al., 2018) and referred to several previous studies that had also used this software to suggest its validity. However, validity of the commercial software is unclear, as it should be noted that none of the studies that were referenced examined or reported the data processing involved.

Test re-test reliability was performed in one study to examine their eye movement outcomes (Cochrane et al., 2019), but it was only performed in healthy controls. Results showed that their eye tracker (I-Portal, Neuro Kinetics Inc.) had poor to moderate reliability [0.02 to 0.71 inter-class correlation coefficient (ICC)] for saccadic accuracy and smooth pursuits, but saccadic latencies and optokinetic gains had better reliability (0.57–0.74 ICC).

### Outcome Measures

Reviewed studies provided outcomes on saccadic (*n* = 12), fixation (*n* = 3), smooth pursuit (*n* = 13), and nystagmus (*n* = 4) eye movements, and there were a plethora of outcomes reported for these eye movements (Table 2, Supplementary Table 1). However, the majority of the reviewed studies did not define their eye movement classifications (i.e., no thresholds or criteria for eye movement detection and measurement). Several studies (Maruta et al., 2010b; Contreras et al., 2011; Cifu et al., 2015; Diwakar et al., 2015; DiCesare et al., 2017; Wetzel et al., 2018; Stuart et al., 2019b) did provide some details regarding definitions but these substantially varied between the studies (Table 2, Supplementary Table 1). Three studies provided no outcomes specific to traditional eye movements (Suh et al., 2006; Murray et al., 2014; Howell et al., 2018), but instead reported on novel outcomes of “Gaze Stabilization” (a fixation measure), “Eye Skew” (an asymmetry measure), and “Oculomotor error” (a smooth pursuit measure) that authors developed for their individual studies. Overall, reporting of possible eye movement outcomes from the eye-tracking devices substantially varied between studies.

### Interpretation of Outcomes

Eye movements (saccades, smooth pursuits, fixations etc.) were generally impaired in mTBI compared to controls or baseline tests, regardless of acute or chronic mTBI status (Table 3). Yet, the influence of mTBI on specific outcomes was inconsistent. For example, several studies found deficits in saccades in people with mTBI during anti-saccadic tests (Johnson et al., 2015a,b; Balaban et al., 2016; DiCesare et al., 2017; Hoffer et al., 2017; Murray et al., 2017; Webb et al., 2018), whereas others found no differences (Wetzel et al., 2018; Cochrane et al., 2019; Kelly et al., 2019). Studies that did not find differences, however, may have been impacted by methodological issues, such as grouping all stages of mTBI together (acute/sub-acute and chronic) to make a larger cohort (Wetzel et al., 2018; Kelly et al., 2019), which limits comparison and understanding of potential deficits at different stages.

**Table 3:** Aims and key findings.

**References**	**Aims**	**Key findings**
Balaban et al. (2016)	Examine oculomotor, vestibular and reaction time reflexes to diagnose mTBI compared to controls.	**Saccades impaired in mTBI** • Increased pro-saccade error rate in mTBI compared to controls • Predictive saccades had significant impairment in mTBI compared to controls, with impaired performance and increased saccadic reaction time latency
Cifu et al. (2015)	Differentiate those with self-reported chronic effects of mTBI from controls	**Saccades and smooth pursuits impaired in mTBI** • Saccades and smooth pursuit eye movements were impaired in mTBI compared with controls • Compared with controls people with mTBI had larger position errors, smaller saccade amplitudes, smaller predicted peak velocities, smaller peak accelerations and longer durations on step-wise displacement targets • Step-wise moving targets were also tracked less accurately and with a smaller primary saccade by those with mTBI compared with controls • Smooth pursuit amplitude was larger and gain was smaller in mTBI compared with controls
Cochrane et al. (2019)	Investigate oculomotor function between mTBI and control college athletes and determine measurement test re-test reliability	**Saccades impaired in mTBI** • Those with mTBI had poor saccadic accuracy and longer response latency compared with controls during horizontal and vertical saccade tasks • No difference between groups during anti-saccade, predictive saccade tasks, or horizontal smooth pursuits • Vertical smooth pursuits were subjectively more difficult for those with mTBI especially at high frequencies • Optokinetic reflex gain was not different between the groups, but 20% of the mTBI subjects were unable to complete due to becoming symptomatic during this test
Contreras et al. (2011)	Investigate the effect of cognitive load on eye-target synchronization in mTBI and controls using non-linear dynamical technique of stochastic phase synchronization	**Smooth pursuits impaired in mTBI** • Horizontal feature of smooth pursuits was not as synchronized in those with mTBI compared with controls • Performing a secondary cognitive task impacted smooth pursuits more in mTBI than controls
DiCesare et al. (2017)	Examined a systematic, automated analysis scheme using various eye-tracking tasks to assess oculomotor function in a cohort of adolescents with acute mTBI symptoms and aged-matched healthy controls	**Fixations and Smooth Pursuits impaired in mTBI** • Greater fixation accuracy error, greater initial fixation error and longer pro-saccade latencies in mTBI compared with controls
Diwakar et al. (2015)	Investigate the neuronal bases for deficient anticipatory control during visual tracking in chronic mTBI patients with persistent symptoms and healthy controls	**Smooth pursuits impaired in mTBI** • Smooth pursuit comparable between groups in continuous tracking condition • In Gap Condition smooth pursuit had larger average radius and had greater negative average phase in mTBI compared with controls • Those with mTBI took longer to respond to target reappearance than controls during gap condition • Time since injury correlated to larger gap average radius in mTBI
Hecimovich et al. (2019)	Determine the diagnostic accuracy of the King-Devick/Eye tracking test in identifying mTBI occurring from game participation and to perform a comparative analysis on saccade and blink counts for each King-Devick card individually and total counts between baseline and post-mTBI	**Saccades impaired in mTBI** • Slower time to completion of task, fewer saccades and more blinks made by those following an mTBI compared with their baseline • Assessment of the number of blinks was most sensitive to mTBI
Hoffer et al. (2017)	Expand previous baseline article findings within several days of injury, through follow-up with further sessions at 7–10 days and 14–17 days. Examine oculomotor, vestibular and reaction time measures to monitor progression of mTBI over the acute and early sub-acute period of time.	**Saccades, smooth pursuits and optokinetic nystagmus impaired in mTBI** • Predictive saccade response differentiated mTBI from controls and was useful to monitor recovery • Pro-saccade performance error rate differentiated mTBI from controls and was useful to monitor recovery • Constant velocity optokinetic nystagmus slow phase gain symmetry for 20°/s stimulation differentiated mTBI from controls and was useful to monitor recovery • Horizontal smooth pursuit absolute velocity gain symmetry differentiated mTBI from controls and was useful to monitor recovery
Howell et al. (2018)	Evaluate objective eye tracking measures among child and adolescent athletes who sustained a mTBI within 10 days of examination and a group of healthy controls	**Eye skew (asymmetry) impaired in mTBI** • Right normalized eye skew along the bottom of the box was greater in those with mTBI compared with controls
Johnson et al. (2015a)	To expand on our previous study by performing a follow-up testing session in the subacute phase of injury for participants recently diagnosed with a mTBI	**Saccades impaired in mTBI** • Longer anti-saccade latencies, greater directional and positional errors, and larger gain in both acute and sub-acute mTBI compared with controls • Average number of self-paced saccades reduced in acute and sub-acute mTBI • Larger primary saccade gain and directional error of memory guided saccades in acute and sub-acute mTBI compared with controls • Some eye movement deficits improved in mTBI from the acute to sub-acute phases of injury
Johnson et al. (2015b)	Examine fMRI in conjunction with a battery of oculomotor tests to simultaneously assess both brain function and eye movements in the acute phase of injury (<7 days post injury) following mTBI	**Saccades impaired in mTBI** • Shorter pro-saccadic error latency, greater anti-saccadic directional and positional errors, and larger anti-saccadic gain in mTBI compared with controls • Average number of self-paced saccades reduced in mTBI • Greater positional error and gain of memory guided saccades in mTBI compared with controls
Kelly et al. (2019)	Test the ability of oculomotor, vestibular, and reaction time (OVRT) metrics to serve as a concussion assessment or diagnostic tool for general clinical use	**Saccades, smooth pursuits and optokinetic nystagmus impaired in mTBI** • Initiation latency was longer for horizontal smooth pursuits at 0.75 and 1.25 Hz, and for vertical pursuits at 0.5 and 0.75 Hz in mTBI compared with controls • Reduced position and velocity gain in horizontal pursuits at 1.25 Hz in mTBI compared with controls • No group difference in random horizontal or vertical saccades, or anti-saccadic, or predictive saccade tests • Percentage of saccade velocities below a normative velocity threshold was higher in mTBI compared with controls during simple reaction time task • Horizontal optokinetic nystagmus response (gain/velocity of slow-phase nystagmus) was reduced in mTBI compared with controls • During high-speed optokinetic nystagmus test; more nystagmus gain asymmetry and higher variability of gain velocity in mTBI than controls • Fast-phase nystagmus area reduced in mTBI compared with controls
Maruta et al. (2010b)	Determine whether performance variability during predictive visual tracking can provide a screening measure for mTBI	**Smooth Pursuit impaired in mTBI** • Poorer visual tracking than controls in mTBI, with large saccadic intrusions and low-velocity gains • Variability measures were correlated with a number of neuroimaging measures. • Smooth pursuit radial and tangential error variability correlated with frontal white matter track integrity and cognitive function in mTBI and controls • Gaze error variability correlated with attention and working memory measures
Maruta et al. (2016)	Characterize cognitive deficits of adult patients who had persistent symptoms after a mTBI and determine whether the original injury retains associations with these deficits after accounting for the developed symptoms that overlap with post-traumatic stress disorder and depression	**Smooth Pursuit impaired in mTBI** Increased smooth pursuit gaze position error variability in mTBI compared with controls after an attention demanding task
Maruta et al. (2017)	Characterize and compare frequency-dependent smooth pursuit velocity degradation in normal subjects and patients who had chronic post mTBI symptoms, and also examine cases of acute mTBI patients	**Smooth pursuit impaired in mTBI** • Reduced horizontal smooth pursuit gain at 0.4 Hz in chronic mTBI compared with controls • No significant difference in smooth pursuit gain at 0.33 or 0.67 Hz in acute and chronic mTBI and controls
Maruta et al. (2018)	Assess changes between pre- and within-2-week post-mTBI performances and explore their relationships to post-mTBI symptomatology	**Smooth pursuit impaired in mTBI** • Horizontal smooth pursuit gain was reduced from baseline following an mTBI • Changes in smooth pursuits with mTBI related to cognition, specifically memory-attention, and physical symptoms
Murray et al. (2014)	Measure the differences in oculomotor control between athletes post mTBI and athletes without concussion during an active balance control task	**Gaze control impaired in mTBI** • Greater gaze deviations from center in mTBI compared with controls
Murray et al. (2017)	Investigate and compare gaze stability between a control group of healthy non-injured athletes and a group of athletes with mTBI 24–48 h post-injury	**Saccades impaired in mTBI** • Greater gaze resultant distance, pro-saccadic errors and horizontal velocity in mTBI compared with controls
Stuart et al. (2019b)	Validate a velocity-based algorithm for saccade detection in infrared eye-tracking raw data during walking (straight ahead and while turning) in people with mTBI and healthy controls	**Saccades can be measured in mTBI while walking** • Developed algorithm accurately detected and classified saccades while walking and turning in mTBI and controls
Suh et al. (2006)	Examined whether those with mTBI would have impairments in prediction during target blanking, and if deficits in eye movement correlated to cognitive deficits.	**Smooth pursuits impaired in mTBI** • Time to first saccade was shorter and intra-individual variability was greater in mTBI compared to controls • Those with mTBI had more ocular motor errors before and during blanking than controls • Ocular motor error variability was also greater in mTBI compared to controls, particularly during blanking • Cognitive outcomes significantly correlated to smooth pursuit outcomes in mTBI
Webb et al. (2018)	Evaluate pro- and anti-saccades in mTBI at an early stage (<6 days) after their injury and at a follow-up assessment.	**Saccades impaired in mTBI** • Pro-saccadic reaction time and gains were not different between mTBI and controls at initial assessment or follow-up • Pro-saccadic directional errors were significantly different between mTBI and controls • Anti-saccades reaction time longer in mTBI than controls at initial assessment but not at follow-up • Anti-saccadic directional errors were greater and gains were lower in mTBI than controls at initial assessment and follow-up
Wetzel et al. (2018)	To identify which visual tasks and measurement parameters are most sensitive in patients with symptoms following mTBI.	**Saccades, fixations, and smooth pursuits impaired in mTBI** • Shorter inter-saccadic interval duration in mTBI compared with controls • Lower absolute saccadic amplitudes and average forward saccadic amplitudes in mTBI compared with controls • Higher absolute fixation velocity and longer overall fixation duration in mTBI compared with controls • Longer regression durations in mTBI and longer forward saccadic durations • More fixations and regressions per line when reading in mTBI compared with controls • Shorter mean fixation times in mTBI compared with controls • Lower weighted smooth pursuit gains in mTBI compared with controls

Other notable methodological limitations were found in the reviewed studies that may impact outcome interpretation. Studies examined the same eye movements but with slightly different protocols. For example, smooth pursuits were examined with a range of frequencies (0.1–1.25 Hz) and the visual stimulus (e.g., colored dots or shapes on a computer or LED board) used for eye movement tasks varied across all studies (Table 2). Although studies reported eye movement outcomes and discussed the relationships between deficits and underlying cognitive or motor impairments due to mTBI, only two studies (Maruta et al., 2010b, 2018) correlated eye movement outcomes with symptoms or other tests for these or other (e.g., age, gender, depression state etc.) relevant features. None of the reviewed articles controlled for the impact of cognition or basic visual function (visual acuity or contrast sensitivity) on eye movements, and only one study (Stuart et al., 2019b) reported basic visual function scores. Many of the studies did not assess cognition and similarly many only reported that they excluded subjects based on visual function (i.e., eye chart screening or self-reported questionnaires) but provided no scores or results to verify this (Suh et al., 2006; DiCesare et al., 2017; Murray et al., 2017; Howell et al., 2018; Webb et al., 2018; Wetzel et al., 2018; Cochrane et al., 2019; Kelly et al., 2019). None of the studies provided any information on the use of corrective eye wear by the participants during the eye-tracking assessments, with several articles reporting that subjects had “normal or corrected to normal vision,” but it was unclear if individuals with vision correction were included in reported results.

### Summary of Common Study Features

In order to refine the information provided in our detailed tables (Tables 1, 2) a brief overview of the most common features of the reviewed studies is presented below;

- The majority of the reviewed studies that provided mTBI diagnostic criteria involved a clinician with experience of sports injuries.
- Included participants tended to be free of previous mTBI, musculoskeletal, cognitive, emotional, visual, or neurological issues.
- Eye-tracker sampling frequency of 500 Hz.
- Seated/static testing using computer screens to provide visual stimuli
- The most common eye-tracker metrics were based upon the latency, gain, velocity, duration, or positional error rates of the specific eye movement assessments, with the velocity of eye movements being the most prominent outcome reported across studies.

## Discussion

This review examined 22 studies that reported quantified eye movements in healthy controls and mTBI subjects. We reviewed; (i) how eye-movements were measured; (ii) reported eye-movement outcomes and their definitions; and (iii) differences reported between mTBI and controls in eye-movement outcomes. Across all of the reviewed studies there was a lack of basic methodological reporting, as there was little consensus or reporting of participant mTBI classification (acute/sub-acute or chronic), demographic characteristics (age, sex, time since injury etc.) and inclusion or exclusion criteria. This limits the generalizability of results to mTBI populations and influences the reproducibility of the methods and results of the reviewed studies. Despite these limitations, this review has demonstrated that eye-movement measurement in mTBI is emerging, but further work is required to establish the validity and reliability of instrumentation and methods to derive eye-movement outcomes, as well as the nature of eye-movement impairments in mTBI.

### Instruments

There is currently no “gold standard” instrument for eye-movement measurement, which is likely the reason for the numerous different instruments used in the reviewed studies. The majority of studies used static infra-red eye-tracking devices in constrained seated activities (e.g., chin rest in place in front of a computer screen), but several studies did show progression to unconstrained dynamic eye-tracking protocols (Maruta et al., 2010b, 2016; Stuart et al., 2019b). High resolution (>100 Hz) mobile eye-tracking during functional activities (e.g., walking or standing) may provide deeper understanding of the impact that subtle eye-movement deficits may have on those with mTBI.

Within the reviewed studies eye-tracking instrumentation sampling frequency substantially varied, which impacts on instrument validity. For example, accurate saccadic detection requires a minimum of 50 Hz and 200 Hz to accurately measure saccadic durations (Andersson et al., 2010; Leube et al., 2017). However several of the reviewed studies only had high enough sampling frequency (60 Hz) to detect saccades (Andersson et al., 2010) but not to accurately measure all of the reported features (Johnson et al., 2015a,b; DiCesare et al., 2017), which limits understanding of subtle deficits that may be missed as a result.

Importantly, clear evidence of the validity and reliability of instrumentation is essential for confidence in reported outcomes. We found that the reviewed studies did not adequately address this, with no studies reporting the validity or reliability of their instrumentation, and four studies inadequately reporting eye-movement outcome validation. Reporting the validity and reliability of eye-tracking instruments is advocated due to the influences of technological [e.g., parallax and calibration error (Pelz and Canosa, 2001; Nystrom et al., 2013)] and physiological [e.g., head or body movement (Zhu and Ji, 2005; Marx et al., 2012)] factors that can impact measurement. Generally, our review revealed a lack of detail regarding instrument design (e.g., binocular, monocular etc.), calibration procedures, sampling frequencies, control for artifact movement etc. To improve the quality of research in this emerging area, there is a need for reporting of the validity and reliability of instruments used to measure eye-movements in mTBI.

### Outcomes

At present there are also no “gold standard” algorithms or definitions for the detection and measurement or reporting of eye-movements (Larsson et al., 2015; Stuart et al., 2019a). This may explain why many of the reviewed studies did not provide definitions for their reported eye-movement outcomes and why reported definitions lacked consensus. As a result, velocity thresholds for saccade detection varied from 20 to 240°/s, with high speeds used for more dynamic tasks (e.g., walking) to rule out the influence of vestibular-ocular reflexes on outcomes. Similarly, eye-movement assessment protocols varied between studies, with different smooth pursuit frequencies, various visual stimuli, overlap or no-overlap designs, different amplitudes of step targets, or speeds and durations of projected dot patterns. The definition of eye-movement outcomes and the protocol used to derive outcomes impacts the generalizability of findings, as valuable information may be discarded or irrelevant data included depending on the thresholds set or the stimulus used. For example, a velocity-based algorithm with a 240°/s threshold will detect saccades over ~5° (Holmqvist et al., 2011), but below this data would be classified as a fixation. However, depending on the specific aims of the study, this algorithm may not be relevant or may not provide an accurate portrayal of performance. This was evident within the reviewed studies where different frequencies of smooth pursuit examination led to studies not finding deficits in mTBI on the same testing paradigms, such as continuous tracking where Diwakar et al. (2015) found no difference at 0.4 Hz but Kelly et al. (2019) found a difference at 1.25 Hz. Creating a gold-standard for eye-movement outcome detection and measurement reporting is challenging due to the variations in instrumentation and different protocols used. Nonetheless, based on the findings within this review, we feel it is necessary for consensus to be adopted for mTBI literature. Such consensus should include the reporting of eye-movement definitions and use of standardized methods [e.g., internationally recognized anti-saccade protocol Antoniades et al., 2013 or algorithms for comprehensive smooth pursuit evaluation (Larsson et al., 2015)] for examining eye-movements.

Within the reviewed studies there was a wide range of eye-movement outcome measures reported, which highlighted the emerging and exploratory nature of eye-movement measurement in mTBI. The majority of the reviewed studies examined smooth pursuits or saccades, with few studies examining fixation and optokinetic nystagmus eye movement outcomes. Focus on smooth pursuits and saccades is likely due to the large amount of underpinning neural regions involved in performance (Ventura et al., 2016), with saccades and pursuits sharing very similar functional architecture within the central nervous system (Krauzlis, 2004). Fixations and saccades have an intimate relationship, as fixations are the pauses in between saccades (Krauzlis et al., 2017), and similarly smooth pursuits could be classified as fixations as they are pauses on moving objects, which may be the reasons why fixations tended to be overlooked within assessments in mTBI. Alternatively, nystagmus eye movements may not have been examined in many studies due to difficulties in monitoring this type of eye movement with infrared eye-trackers. For example, measurement is influenced by head position (Pettorossi et al., 2011), and electro-oculography is usually used due to accuracy and high sampling frequency requirements (Haslwanter and Clarke, 2010). In addition, stimuli that evoke sustained nystagmus eye movements commonly exacerbate mTBI-related symptoms, with reviewed studies reporting having to stop testing, as a result, which led to reduced cohort sizes for further investigation (Cochrane et al., 2019). Similarly, many of the reviewed studies had small (*n* < 30) mTBI cohorts and as a result the number of outcomes reported may lead to inappropriate statistical analysis or reporting due to the number of performed statistical comparisons (e.g., Type I or II statistical error; von Der Malsburg and Angele, 2017). Similar to other behavioral measures (e.g., gait; Verghese et al., 2007), use of data reduction techniques, such as principle component or factor analysis, in future studies may help to reduce the risk of inappropriate outcome reporting by developing relevant eye-movement domains or factors for further analysis.

Despite the huge number of outcome measures reported, many of the outcomes were reported in a task-dependent manner. For example, static seated eye-movement assessments tended to comprehensively report many outcomes from long testing protocols, whereas dynamic standing or walking tasks tended to report a small number of largely saccadic outcomes (e.g., saccade number, velocity, amplitude; Murray et al., 2014, 2017; Stuart et al., 2019b). This task-dependent reporting of eye-movement outcomes likely stems from the complexity of data processing and analysis with increasingly dynamic tasks (Zhu and Ji, 2005; Stuart et al., 2014b, 2019a). Unlike controlled static seated assessments, dynamic tasks introduce other factors (e.g., head movement, vestibular-ocular reflexes, lighting conditions of testing) that can impact recordings and need to be controlled for as these factors have been demonstrated to influence eye-tracking outcomes (Stuart et al., 2017). Comparison of several studies that reported saccade velocity indicated that there may be task-dependent mTBI impairments. For example, seated studies reported reduced saccadic velocity in mTBI compared to controls (Cifu et al., 2015; Cochrane et al., 2019; Kelly et al., 2019), whereas dynamic studies reported the opposite (i.e., greater saccadic velocity in mTBI; Murray et al., 2017). However, due to the limited number of studies available for review and methodological variations, definitive conclusions cannot currently be drawn. This confirms the need to appropriately quantify eye-movements in mTBI during a range of different tasks to uncover impairments that are relevant to performance of “real-life” activities.

### Interpretation of Outcomes

Generally, the reviewed studies showed that saccades, smooth pursuits, fixations and nystagmus were impaired in mTBI compared to controls. However, eye-movement outcome interpretation was complicated by many methodological limitations, particularly the inconsistent or lack of reporting of basic demographic and mTBI-related information (e.g., mTBI diagnosis criteria, time since injury etc.). There is currently no universally accepted definition or diagnostic criteria for mTBI (Carroll et al., 2004; Management of Concussion/mTBI and Working Group, 2009; Mccrory et al., 2017; Eisenberg and Mannix, 2018; Voormolen et al., 2018; Chancellor et al., 2019), which is likely the reason why the majority of studies did not describe the criteria that were met or how/who provided an mTBI diagnosis. However, many of the reviewed studies also provided very little demographic information regarding their participants, with some studies not reporting basic features such as participant sex or specific time since mTBI (e.g., days, weeks, months, or years since injury). Lack of reporting accompanied by variable inclusion and exclusion criteria between studies makes the generalization across the literature difficult, and may explain some of the conflicting reports of specific eye-movement deficits (i.e., reports of anti-saccadic impairment was variable). Lack of a standardized mTBI diagnosis criteria and reporting of basic features makes interpretation of outcomes complex (King, 2019), therefore studies should report these features as fully as possible to aid understanding.

There were a vast number of outcomes being recorded and reported, which ranged from relatively standard (e.g., pro-saccades, anti-saccades, smooth pursuits etc.) to novel [e.g., eye skew (Howell et al., 2018), gaze stabilization (Murray et al., 2014), oculomotor error (Suh et al., 2006)] outcomes. Development and application of novel eye-movement outcomes may allow deficits to be uncovered that may otherwise not be found (Harezlak and Kasprowski, 2018). However, the lack of reporting on novel outcome validation and data processing in the reviewed studies limits the generalizability of results and does not allow replication in other cohorts. Our findings suggest that validity and reliability assessment of novel outcome measures should be reported alongside standardized eye-movement outcomes from traditional testing batteries, which would situate novel outcomes in the context of traditional measures to aid interpretation.

We were surprised that only two studies (Maruta et al., 2010b, 2018) assessed for cognitive function and only one study reported data on visual function (Stuart et al., 2019b), with other studies only reporting that participants had “normal or corrected to normal vision” (Suh et al., 2006; DiCesare et al., 2017; Murray et al., 2017; Howell et al., 2018; Webb et al., 2018; Wetzel et al., 2018; Cochrane et al., 2019; Kelly et al., 2019). Eye-movements are underpinned by cognitive processes (Hutton, 2008; Mele and Federici, 2012), even from an early stage before the automatic bottom-up cascade of visual processing occurs (Baluch and Itti, 2011). Cognitive function was shown to relate to eye-movement performance in mTBI in several reviewed studies (Suh et al., 2006; Maruta et al., 2010b, 2018), with cognitive deficits leading to abnormal performance on quantified eye-movement examinations. Eye-movements may therefore be a proxy for cognitive function in mTBI, but without assessment and controlling for cognitive function within eye-movement analysis these links may be missed (Stuart et al., 2014a). Similarly, impairments in basic visual functions, such as visual acuity and contrast sensitivity, have been found to lead to abnormal eye-movement performance (Williams et al., 1995; Gottlob et al., 1996; Palidis et al., 2017). Visual acuity deficits can be corrected with prescription glasses or contact lenses (Sloan, 1951), however the reviewed studies provided no details regarding whether participants used visual correction during testing. This is important as visual correction, through glasses and contact lenses, can impact infrared eye-tracking due to the refraction of infrared light that is used to detect the location of the participants pupil, which would lead to inaccurate tracking and lost data collection ability (Stuart et al., 2014b, 2016; Fuhl et al., 2016). Age (Munoz et al., 1998), depression (Emslie et al., 1990), and medication use [e.g., opioids (Grace et al., 2010)] have also been implicated in eye-movement performance in various populations. Therefore, the measurement and reporting of basic demographic features, cognitive and visual function is required when investigating eye-movements in mTBI.

### Test Protocols

Previous eye-tracking studies have generally involved static seated eye-movement assessment tasks (Pelz and Canosa, 2001; Maruta et al., 2010a), which have provided valuable information regarding potential deficits in mTBI compared to controls. However, while these experiments allow for complete experimental control, they lack functional validity because eye-movements in the real-world are goal-oriented (Salverda et al., 2011) and often occur during co-ordination of multiple motor, cognitive and visual processes (Hayhoe et al., 2003; Hayhoe and Ballard, 2005; Ventura et al., 2016). Although we found that segmentation of individual eye-movement features revealed some eye-movement impairments in mTBI, inconsistencies in reported deficits may be due to compensatory mechanisms (i.e., using additional attentional resources to improve performance of eye-movement tasks) similar to those found in aging research (Wiegand et al., 2014). Whereas, when individuals are required to complete complex real-world tasks (e.g., walking, turning, balancing etc.), that simultaneously involve motor, cognitive and visual processes, deficits may become prominent as they may be unable to compensate, consistent with results from the dual-task literature in mTBI (Cicerone, 1996; Howell et al., 2013). Future studies should therefore robustly examine eye-movements within mTBI during a range of static and dynamic tasks to further understand the functional impact of deficits.

Many previous studies of eye-movements in mTBI have incorporated subjective, clinical assessments that can be performed is any environment, such as the VOMS (Mucha et al., 2014) or King-Devick reading test (Leong et al., 2015; Galetta et al., 2016; Walsh et al., 2016). These clinical tests provide valuable information concerning the aggravation of mTBI-related symptoms when performing eye-movements (Capó-Aponte et al., 2018), but provide limited quantifiable information to measure deficits or monitor recovery. These subjective or symptom-based eye-movement examinations provide only global performance measures and symptom scores following an mTBI, but results cannot highlight subtle changes in performance and may be limited due to the reliance on self-report by those with mTBI (Lovell et al., 2002; Heitger et al., 2007). In contrast, the 22 studies included in this review examined eye-movements using quantitative eye-tracking technology that can provide information related to subtle changes in eye-movements, and link them to specific mechanisms behind impairments through experimental manipulation. Future adoption of quantitative eye-tracking technologies within clinical practice will allow eye-movement examination to become standardized and may detect deficits that could be missed with traditional clinical techniques.

## Conclusion

Aspects of eye-movements are impaired following an mTBI, as demonstrated by the reviewed studies that quantified impairments in either saccadic, smooth pursuit, fixation or nystagmus eye movements. While there is evidence from these studies, it is not yet strong enough to adopt quantitative eye-movement assessment within clinical practice. This review has highlighted that methodological issues across the current literature limit the understanding and generalizability of the reported findings in mTBI. There is a need for consensus on methods used and reporting of eye movement data, including eye-movement instruments, data collection procedures, processing algorithms and analysis methods are required. Comprehensive reporting of demographics, mTBI-related features, and confounding variables are also recommended for future work in this area (Table 4). Development and implementation of a standardized approach to quantitative eye-movement examination will ensure accurate and appropriate data interpretation. This will allow robust evidence to be established that can be implemented in future clinical practice.

**Table 4:** Recommendations for future research.

**Recommendations for future research examining eye-movements in mTBI**
• Comprehensively report demographic data including; age, sex, depression, medication use, time since injury • Report study inclusion and exclusion criteria • Stratify mTBI populations based on time since injury; acute, sub-acute, chronic, post-mTBI syndrome • Report recruitment strategy; hospitals, community, athletes, military etc. • Provide a definition of the mTBI diagnosis or criteria • Report details of instrumentation used to record eye-movements, including; sampling frequency, binocular or monocular, calibration procedure, infrared or video based, desk or head-mounted • Report validity and reliability of eye-movement recording instrumentation, data processing algorithms and outcomes • Use an adequately powered sample size or data reduction techniques to appropriately report eye-movement outcomes • Define all eye-movement outcome measures • Report whether corrective lenses were used for testing • Routinely examine and control for cognitive and visual function

## Author Contributions

SS wrote the first draft and revisions of the manuscript. SS, LP, and DM designed the search strategy and conducted the literature search. SS and LK designed, implemented, and oversaw the topic area. SS, LP, DM, RP, JC, and LK were involved in interpretation of the data, writing the manuscript and revisions, final approval of the version to be published, and agree to be accountable for all aspects of the work.

### Conflict of Interest

The authors declare that the research was conducted in the absence of any commercial or financial relationships that could be construed as a potential conflict of interest.
