# Supplementary material for: The Measurement of Eye Movements in Mild Traumatic Brain Injury: A Structured Review of an Emerging Area
Source: Front Sports Act Living. 2020 Jan 28;2:5. doi: 10.3389/fspor.2020.00005 (PMC7739790; doi:10.3389/fspor.2020.00005)
Supplement: Supplementary file 1 [file Table_1.DOCX]

**Supplementary Table 1 – Detailed description of testing protocols and eye movement outcome measures**

| Author | Test Protocol | Eye Movement Outcome Measures |
| --- | --- | --- |
| Balaban et al. (2016) | **Static/seated**  - Sat in a lightproof enclosure on a rotary chair, 36inches from the black featureless walls  - Pattern of random moving dots, covered at least 90% of the visual field was projected onto the walls for optokinetic stimuli  - A projected 650nm 3mm laser dot was used for fixation, pursuit and saccade target stimuli  - Predictive saccades had a start less than 50ms after target onset and alternate ± 10° at fixed intervals of 650ms  - Testing repeated 3 times at equivalent intervals over an average of 18 days; only first testing session reported | Saccades  Random Saccades   - Onset latency - Accuracy - Peak velocity - Area under the main sequence relationship   Anti-saccades   - Pro-saccadic errors - Correct Anti-saccades - Anti-saccade latency - Anti-saccade velocity   Predictive saccades   - Prediction timing - Percentage of correct predictions   Self-paced saccades   - Self-paced saccades per second   Smooth Pursuit   - Percentage of saccadic intrusions - Initiation time   Optokinetic Nystagmus   - Left and right gain - Asymmetry for nystagmus beats   Gaze horizontal   - Vertical peak and average slow phase velocity |
| Cifu et al. (2015) | **Static/seated**  - Sat 75cm from a computer monitor  - Darkened room  - White target presented on black background  - Stimuli consisted of random, unpredictable step target movements and smooth pursuit paradigms in horizontal and vertical directions  - Subjects asked to close eyes between trials for rest | Saccade   - Time - Location - Response latency - Amplitude - Direction - Duration - Peak Velocity - Peak Acceleration - Peak Deceleration - Saccadic gain - Positioning error   Fixation   - Root mean square of velocity - Mean velocity - Absolute velocity - Orientation - Semi-major dimensions - Semi-minor dimensions - Ares of elliptical contour (deg²) - Position variance   Smooth Pursuit   - Number of saccadic intrusions - Saccadic intrusion amplitude - Pursuit gain; ratio between weighted mean eye velocity and target velocity (without inclusion of corrective saccades) |
| Cochrane et al. (2019) | **Static/seated**  - 25-30 minute test battery  - Red laser dot on wall  - Horizontal tests completed before vertical  - *Predictive saccades*  - Followed a target as moved horizontally in a fully predictable stepwise manner  - 24 steps in total, beginning with 5 random steps  - 10º right and left, with hold time of 0.65s  - 18 target steps for prediction analysis  - *Random horizontal and vertical saccades*  - Followed a target as it stepped randomly to right and left, then up and down  - 30 target steps within -30 to 30º  - Transitions 1.1-2s and initial target range was -22º to 24º  - *Smooth Pursuit*  - Followed a target moving sinusoidal horizontal and vertical at 0.01, 0.02, 0.04, 1.00Hz (horizontal) or 0.75Hz (vertical)  - ±10º peak to peak  - *Anti-saccades*  - Initial focussed on central target, then random movement of target to left or right, asked to generate a saccade in opposite direction at same size as step target, and hold gaze there  - 16 trials  - *Optokinetic Nystagmus*  - Watched a random dot pattern projected on the enclosed wall that moved counter clockwise then clockwise at 20º/s or 60º/s for 20s  - 4s between change in direction | Saccade   - % of predictive saccades - 1^st^ predictive saccade - Random horizontal and vertical saccades: primary saccade accuracy, mean final accuracy, mean latency - Anti-saccades: % error   Smooth Pursuit   - Average velocity gain - Average position gain   Optokinetic Nystagmus   - Gain during first 20s of task |
| Contreras et al. (2011) | **Static/seated**  - Sat 40cm from a computer screen  - Darkened room  - Smooth Pursuit  - Tracked a target (red circle, 0.2º diameter)  - Trajectory of 10º radius at a rate of 0.4Hz in clockwise direction  - Trials lasted 30s | Smooth Pursuit   - Eye velocity error (º/s) - Number of saccadic intrusions |
| DiCesare et al. (2017) | **Static/seated**  - Sat 65cm from computer screen  - Tracked a dark grey circular target (4cm diameter) with a black cross hair in the centre (1cm diameter), against a light grey background  - Total of 18 tests  - 2 x 30s Pro-saccade task  - Target randomly stepped along a horizontal line, asked to keep eyes focussed on centre of cross-hair in target  - step magnitude 4º and 10º  - Static after each step for 1-2s  - 2 x 30s of self-paced saccade task  - Two targets on screen, not moving, at 50% height of screen, 8º apart  - Asked to look back and forth between targets left and right as quickly and accurately as possible  - 2 x smooth pursuit task at 90, 180 and 360º/s  - Target moved sinusoidal wave left to right  - Asked to keep eyes focussed on crosshair in centre of the target  - Speeds block randomised  - Initial fixation for 2s on target before task | Saccades   - Pro-saccade latency average - Pro-saccade average velocity - Total number of self-paced saccades - Self-paced saccade average velocity   Fixation   - Error   Smooth Pursuit   - Phase lag - Average Velocity - Gaze angular velocity average |
| Diwakar et al. (2015) | **Static/seated**  - Sat 125.4cm from a computer screen  -*Smooth Pursuit*  - Tracked a red disk-shaped target (0.9º diameter) on a black background  - Moved 10º at 0.4Hz clockwise in a circle  - 10 trials, 2.5s per trial (25s in total)  - *Gap condition Smooth Pursuit*  - Same as above, but target visible for random time between 1250-3250ms, then disappeared for 208ms, 312ms and 416ms before reappearing  - Single practice block before task | Smooth Pursuit   - Average radius of the gaze trajectory - Average phase error - Variability of tangential error - Variability of radial error - Saccade frequency |
| Hecimovich et al. (2019) | **Static/seated**  - One trial of King-Devick eye-tracking test  - Sat 60cm from screen  - Read numbers aloud left to right, from top to bottom  - 2 minute task | Saccades   - Number   Blinks   - Number |
| Hoffer et al. (2017) | **Static/seated**  - Sat in a lightproof enclosure on a rotary chair, 36inches from the black featureless walls  - Pattern of random moving dots, covered at least 90% of the visual field was projected onto the walls for optokinetic stimuli  - A projected 650nm 3mm laser dot was used for fixation, pursuit and saccade target stimuli  - Predictive saccades had a start less than 50ms after target onset and alternate ± 10° at fixed intervals of 650ms  - Testing repeated 3 times at equivalent intervals over an average of 18 days; all testing sessions reported and comparison across time | Saccades  Random Saccades   - Onset latency - Accuracy - Peak velocity - Area under the main sequence relationship   Anti-saccades   - Pro-saccadic errors - Correct Anti-saccades - Anti-saccade latency - Anti-saccade velocity   Predictive saccades   - Prediction timing - Percentage of correct predictions   Self-paced saccades   - Self-paced saccades per second   Smooth Pursuit   - Percentage of saccadic intrusions - Initiation time   Optokinetic Nystagmus   - Left and right gain - Asymmetry for nystagmus beats   Gaze horizontal   - Vertical peak and average slow phase velocity |
| Howell et al. (2018) | **Static/seated**  - Sat 55cm from screen, chin rest in situ  - Watched a 220s video clip from internet sources  - Video displayed on screen in 1/9^th^ size and moving clockwise along edge of screen | Eye skew  Normalised eye skew  Eye movement variance ratio  Eye distance |
| Johnson et al. (2015a) | **Lying down in MRI machine**  **-** Saccades performed in horizontal direction  - Eye fixation  - Reflexive saccades  - Anti-saccades  - Memory-guided saccades  - Self-paced saccades  - Circular and sinusoidal smooth pursuit | Saccades  Anti-saccade   - Latency - directional error - primary saccade gain - gain of final eye position - position error   Self-paced saccade   - number - gain of final eye position - Position error   Memory-guided saccade   - directional error - primary saccade gain - gain of final eye position - Position error |
| Johnson et al. (2015b) | **Lying down in MRI machine**  - *Eye fixation*  - Look at central target 30s  - *Reflexive saccades*  - 44 green circular targets (0.75º diameter) presented one by one at random positions 0º, 5º, 10º and 15º from centre on black background  - Intervals for presentation were random between 1-1.6s  - Asked to look at target as quickly and accurately as possible  - *Anti-saccades*  - 32 green circular targets (0.75º diameter) presented one by one at random positions 5º and 10º from centre on black background  - Target presentation and timing were randomised, as above  - Left and right trials 5-10º  - Asked to look in opposite direction to mirrored position as quickly and accurately as possible  - *Memory guided saccades*  - 6 sequences of green circular targets that moved to pre-defined horizontal positions every second for 3 times in one sequence  - Target remained in each position for 2s  - Each sequence displayed 5 times during encoding phase  - Asked to repeat back sequence on blank black background for retrieval phase  - *Self-paced saccades*  - 2 green circular targets displayed at ±15º from centre on black background  - 30s trial  - Asked to look back and forth between two targets as quickly and accurately as possible  - *Smooth Pursuit*  - Asked to track centre of a moving target  - Predictable horizontal sinusoidal pattern tracking with peak velocity 40º/s  - Circular tracking with 12º visual arc radius and tangential velocity at 30º/s  - Random tracking in horizontal direction; mean peak velocity 60º/s  - Each test lasted 40s | Saccades  Reflexive saccades   - Latency - Velocity - mean absolute position error   Anti-saccades   - Numbers of directional errors - latency - percentage of directional errors - correction time for the erroneous saccades - Velocity - Mean absolute position errors   Memory guided saccades   - Numbers of directional errors - Mean absolute position errors   Self-paced saccades   - Number of saccades - Mean inter saccadic interval   Smooth Pursuit   - Average eye peak velocity - Tracking lag   Fixation   - Mean absolute position error |
| Kelly et al. (2019) | **Static/seated**  - Sat in front of wide white reflective screen in dimly lit room  - *Smooth Pursuit*  - 6 tasks; 3 horizontal and 3 vertical  - Asked to track a laser-projected target in sinusoidal velocity across ±10º at 0.1, 0.75 and 1.25Hz (horizontal), and 0.1, 0.5 and 0.75Hz (vertical)  - *Saccades*  - Targets displayed at random locations along horizontal and vertical axes  - Predictive saccades: timing to evaluate prediction of movement  - Anti-saccades: asked to look in opposite direction to the target  - *Optokinetic Nystagmus*  - Horizontally moving field of illuminated dots by rotating projector at 23.4º/s or 70.3º/s  - 10s one direction then reversed for 10s | Smooth Pursuit   - Position gain - Velocity gain - Velocity gain asymmetry - Presence of saccadic movement - Latency to initiate SP tracking   Saccades  Predictive saccades   - latency to initiate a saccade - accuracy - peak velocity relative to a normative threshold and as a function of saccade amplitude   Anti-saccades   - latency to initiate a saccade - accuracy - peak velocity relative to a normative threshold and as a function of saccade amplitude   Optokinetic Nystagmus   - Velocity gain - Asymmetry of gain - Area under fast-phase fit |
| Maruta et al. (2010b) | **Static/seated**  - Sat 40cm from computer screen  - head and chin rest in situ  - Tracked target (red circle 0.2º diameter) moving in circular trajectory of 8.5º radius  - 10-12 cycles moving at 0.4Hz  - Black background | Smooth Pursuit   - Standard deviation of radial errors - Standard deviation of tangential errors - Mean phase error - Velocity gains horizontal and vertical |
| Maruta et al. (2016) | **Static/seated**  - Sat with chin rest  - -Sat wearing a stimulus-presentation device integrated with a video-based eye-tracker  - Stimulus presented at 120Hz  - Tracked target at a constant speed of 25.1º/s (0.4Hz) in a clockwise direction along a circular path with a radius of 10º  - Black background  - Normally lit room | Smooth Pursuit   - Saccadic intrusions identified and removed - Standard deviation of radial errors - Standard deviation of tangential errors - Mean radial error - Mean phase error - Velocity gains horizontal and vertical |
| Maruta et al. (2017) | **Static/seated**  -Sat wearing a stimulus-presentation device integrated with a video-based eye-tracker  - Tracked a target that moved clockwise in circular trajectory at 0.33, 0.4 and 0.67Hz with 10º visual angle  - Corresponding target speeds were 21°, 25°, and 42°/s  - Random order of stimulus frequencies  - Testing sequence lasted approximately 5 minutes per stimulus frequency | Smooth Pursuit   - Gain velocity - Phase velocity |
| Maruta et al. (2018) | **Static/seated**  - Sat with chin rest  - Sat wearing a stimulus-presentation device integrated with a video-based eye-tracker- Red target (0.5º diameter) on black background  - Tracked target at a constant speed of 25.1º/s in a clockwise direction along a circular path with a radius of 10º  - Normally lit room  - Test lasted 5mins in total: 2 x 15s recording tests | Smooth Pursuit   - Standard deviation of radial errors - Standard deviation of tangential errors - Mean radial error - Mean phase error - Velocity gains horizontal and vertical |
| Murray et al. (2014a) | **Dynamic/Standing**   - Standing playing a Nintendo WiiFit soccer heading game on Wii balance board | Gaze stabilisation   - Percentage time on centre of screen - Number of gaze deviations (eye movements) from centre of screen |
| Murray et al. (2017) | **Dynamic/Standing**   - Standing playing a Nintendo WiiFit soccer heading game on Wii balance board - 3 trials of 62s - Stimulus (soccer balls on screen) appeared at 0.80s intervals and took 2s to travel horizontally towards participants avatar on screen | Saccades   - Number of pro-saccade errors - Resultant distance (pix) - Mean horizontal velocity (pix/s) - Mean vertical velocity (pix/s) |
| Stuart et al. (2019b) | **Dynamic/Walking**   - Walking back and forth over 10m for 1 minute - 8 laps of a turning course consisting of 45º, 90º and 135º consecutive turns   Completed tasks under single and dual task (auditory stroop) and fast walking conditions | Saccade   - Number |
| Suh et al. (2006) | **Static/seated**  - Seated in a darkened room with bite bar system  - 40cm from computer screen  - 3 blocks of 35 clockwise circular trajectories (7 degree radius 0.4Hz frequency)  - Period of target blanking at 0°, 30°, 135°; random duration 208ms and 938ms | Smooth Pursuit   - Time to first saccade after target blanking - Horizontal oculomotor error - Oculomotor error intra-individual variability - Phase (lead or lag) |
| Webb et al. (2018) | **Static/seated**  - Sat in chair in front of table (775mm height)  - Stimulus board (690 by 470mm) on table top 550mm from participant  - Board had light emitting diodes (LEDs, luminance 48cd/m^2^); multi-coloured and yellow LEDs for fixation in centre of board  - Targets at 15.5º and 20.5º left and right  - Visual stimuli controlled via MATLAB Psychophysics Toolbox extensions (ver. 3.0)  - Initial fixation on centre cross that changed to green (pro-saccade) or red (anti-saccade) to direct participant to move eyes to correct location (Overlap paradigm; central fixation target still visible)  - Targets were presented for 50ms  - Random fore period of 1000-2000ms was used for initial fixation (±1.5° from 500ms)  - Participant asked to move eyes to target as quickly and accurately as possible  - Pro- and anti-saccade performed in separate blocks that were randomly ordered  - 10 trials per block; total of 40 pro-saccades and 40 anti-saccades  - 160 trials in total that lasted 10mins | Anti-saccades  - Reaction time (mean and coefficient of variation)  - Percentage directional errors  - Amplitude gain  - Primary movement direction  Pro-saccades  - Reaction time (mean and coefficient of variation)  - Percentage directional errors  - Amplitude gain  - Primary movement direction |
| Wetzel et al. (2018) | **Static/seated**  - Sat 75cm from computer screen  - *2 point saccades*  - Two targets presented 10º apart  - Asked to move gaze as quickly as possible between targets  - 10s trial  - *Circular smooth pursuit*  - Target moved in circular pattern  - Asked to follow the target  - Rotational period of 0.25s (0.4Hz), amplitude *±9º, peak velocity 22º/s*  *- Horizontal and vertical ramp smooth pursuit*  - Target moved across screen with multiple random segments of different velocities, durations and directions  - 30s trials  - Horizontal: Maximum velocity 16.72º/s, minimum 4.20º/s, weighted average velocity 9.07º/s, mean duration 1.082 ± 0.536s, maximum duration 2.576s, minimum 0.554s  - Vertical: Maximum velocity 14.45º/s, minimum 3.60º/s, weighted average velocity 7.08º/s, mean duration 1.082 ± 0.536s, maximum duration 2.576s, minimum 0.554s  - *Horizontal and vertical step saccades*  - Target jumped horizontally or vertically and target presented as a gap where first stimulus disappeared and then second stimulus appeared  - Randomised for time and position of the jump  - 30s trials  - Horizontal: Mean step size 12.10º± 8.63º, minimum step size 0.94º, maximum 29º, mean position duration 1.16 ± 0.48s, minimum duration 0.616s, maximum 2.086s  - Vertical: Mean step size 7.28º ± 5.13º, minimum step size 0.61º, maximum 17.26º, mean position duration 1.16 ± 0.48s, minimum duration 0.616s, maximum 2.086s  *- Reading saccades*  - 5 texts with 10 lines  *- Memory guided on and off*   - Two types of trials: 1) Pattern shown to the participant and the participant followed the pattern with his or her gaze while it remained illuminated. 2) Pattern shown to participant then disappeared, and the participant tried to repeat the pattern - 19 Target positions 0°, 5°, and 10°, duration, 1.5 second per target position. - Total estimated time: 60s each. Performance measures:   - *Anti-saccade*   - Move eyes in an equal but opposite direction from the actual target position. - 20 Target positions at 2°, 7°, 9°, 12°, and 17°, duration at each target position 1.7s. - Total estimated time: 33s. | Saccade   - Amplitude - Duration - Velocity - Acceleration   Self-paced saccades (reading)   - Forward and regressive saccadic amplitude per line - Primary return sweep amplitude   Memory guided saccades   - Target off and target on hits and misses   Anti-saccades   - Hits and misses   Smooth Pursuit   - Saccadic amplitude - Inter saccadic interval duration - Velocity - Weighted inter saccadic interval velocity - Weighted smooth pursuit gains   Fixation   - Time - Velocity - Reading; forward fixation and regression duration, lines read, fixation and regressions per line. |
